# Comparative genomics of *Fructobacillus* spp. and *Leuconostoc* spp. reveals niche-specific evolution of *Fructobacillus* spp.

**DOI:** 10.1186/s12864-015-2339-x

**Published:** 2015-12-29

**Authors:** Akihito Endo, Yasuhiro Tanizawa, Naoto Tanaka, Shintaro Maeno, Himanshu Kumar, Yuh Shiwa, Sanae Okada, Hirofumi Yoshikawa, Leon Dicks, Junichi Nakagawa, Masanori Arita

**Affiliations:** Department of Food and Cosmetic Science, Faculty of Bioindustry, Tokyo University of Agriculture, 196 Yasaka, Abashiri, Hokkaido 099-2493 Japan; Department of Computational Biology and Medical Sciences, Graduate School of Frontier Sciences, The University of Tokyo, Kashiwa, Chiba Japan; Center for Information Biology, National Institute of Genetics, Mishima, Japan; NODAI Culture Collection Centre, Tokyo University of Agriculture, Tokyo, Japan; Functional Foods Forum, University of Turku, Turku, Finland; Genome Research Center, NODAI Research Institute, Tokyo University of Agriculture, Tokyo, Japan; Department of Bioscience, Tokyo University of Agriculture, Tokyo, Japan; Department of Microbiology, University of Stellenbosch, Stellenbosch, South Africa; RIKEN Center for Sustainable Resource Science, Yokohama, Japan

**Keywords:** *Fructobacillus*, *Leuconostoc*, Comparative genomics, Fructophilic lactic acid bacteria, Niche-specific evolution, Metabolism

## Abstract

**Background:**

*Fructobacillus* spp. in fructose-rich niches belong to the family *Leuconostocaceae*. They were originally classified as *Leuconostoc* spp., but were later grouped into a novel genus, *Fructobacillus*, based on their phylogenetic position, morphology and specific biochemical characteristics. The unique characters, so called fructophilic characteristics, had not been reported in the group of lactic acid bacteria, suggesting unique evolution at the genome level. Here we studied four draft genome sequences of *Fructobacillus* spp. and compared their metabolic properties against those of *Leuconostoc* spp.

**Results:**

*Fructobacillus* species possess significantly less protein coding sequences in their small genomes. The number of genes was significantly smaller in carbohydrate transport and metabolism. Several other metabolic pathways, including TCA cycle, ubiquinone and other terpenoid-quinone biosynthesis and phosphotransferase systems, were characterized as discriminative pathways between the two genera. The *adhE* gene for bifunctional acetaldehyde/alcohol dehydrogenase, and genes for subunits of the pyruvate dehydrogenase complex were absent in *Fructobacillus* spp. The two genera also show different levels of GC contents, which are mainly due to the different GC contents at the third codon position.

**Conclusion:**

The present genome characteristics in *Fructobacillus* spp. suggest reductive evolution that took place to adapt to specific niches.

**Electronic supplementary material:**

The online version of this article (doi:10.1186/s12864-015-2339-x) contains supplementary material, which is available to authorized users.

## Background

Lactic acid bacteria (LAB) are found in a variety of environments, including dairy products, fermented food or silage, and gastrointestinal tracts of animals. Their broad habitats exhibit different stress conditions and nutrients, forcing the microbe to develop specific physiological and biochemical characteristics, such as proteolytic and lipolytic activities to obtain nutrients from milk [1], tolerance to phytoalexins in plants [2], or tolerance to bile salts to survive in the gastrointestinal tracts [3]. *Fructobacillus* spp. in the family *Leuconostocaceae* are found in fructose-rich environments such as flowers, (fermented) fruits, or bee guts, and are characterized as fructophilic lactic acid bacteria (FLAB) [4–6].

The genus *Fructobacillus* is comprised of five species: *Fructobacillus fructosus* (type species), *F. durionis, F. ficulneus, F. pseudoficulneus* and *F. tropaeoli* [6, 7]. Four of the five species formerly belonged to the genus *Leuconostoc*, but were later reclassified as members of a novel genus, *Fructobacillus*, based on their phylogenetic position, morphology, and biochemical characteristics [8]. *Fructobacillus* is distinguished from *Leuconostoc* by the preference for fructose over glucose as the carbon source and the need for an electron acceptor (e.g. pyruvate or oxygen) during glucose assimilation. *Fructobacillus* is further differentiated from *Leuconostoc* by the production of acetic acid instead of ethanol when glucose is metabolized. We previously compared these microorganisms with special attention to the activities of alcohol and acetaldehyde dehydrogenases; *Fructobacillus* lacks the bifunctional acetaldehyde/alcohol dehydrogenase gene (*adhE*) [9] and its enzyme activities. They are the only obligately heterofermentative LAB without *adhE* to date, suggesting that niche-specific evolution occurred at the genome level. Recent comparative genomic studies also revealed niche-specific evolution of several LAB, including vaginal lactobacilli and strains used as dairy starter cultures [10–12].

This is the first study to compare the metabolic properties of the draft genome sequences of four *Fructobacillus* spp. with those of *Leuconostoc* spp., with a special focus on fructose-rich niches. Results obtained confirm the general trend of reductive evolution, especially metabolic simplification based on sugar availability.

## Methods

### Bacterial strains and DNA isolation

*Fructobacillus fructosus* NRIC 1058^T^, *F. ficulneus* JCM 12225^T^, *F. pseudoficulneus* DSM 15468^T^ and *F. tropaeoli* F214-1^T^ were cultured in FYP broth (l^−1^: 10 g D-fructose, 10 g yeast extract, 5 g polypeptone, 2 g sodium acetate, 0.5 g Tween 80, 0.2 g MgSO_4_ . 7H_2_O, 0.01 g MnSO_4_ . 4H_2_O, 0.01 g FeSO_4_ . 7H_2_O, 0.01 g NaCl; pH 6.8) at 30 °C for 24 h. Genomic DNA was isolated by the method of a combination of phenol/chloroform and glass beads as described previously [13].

### Draft genome sequencing and *de novo* assembly

Whole-genome sequencing was conducted by Illumina Genome Analyzer II system, with insert length of about 500 bp. Total 6,060,140, 1,904,646, 2,474,758 and 13,680,640 reads with average lengths of 60 to 91 bp were obtained from *F. fructosus* NRIC 1058^T^, *F. ficulneus* JCM 12225^T^, *F. pseudoficulneus* DSM 15468^T^ and *F. tropaeoli* F214-1^T^, respectively. *De novo* assembly using the Velvet Assembler for short reads with parameters optimized by the VelvetOptimizer (Version 1.2.10) [14] resulted in 57, 28, 15 and 101 contigs each (Length: 1,489,862, 1,552,198, 1,413,733 and 1,686,944 bp; N_50_: 89,458, 226,528, 283,981 and 226,443 bp). The *k-*mer sizes for the strains were 81, 45, 51, 63 bp each. The genome was annotated using the Microbial Genome Annotation Pipeline (MiGAP) [15] with manual verification. In the pipeline, protein coding sequences (CDSs) were predicted by MetaGeneAnnotator 1.0 [16], tRNAs were predicted by tRNAscan-SE 1.23 [17], rRNAs were predicted by RNAmmer 1.2 [18], and functional annotation was finally performed based on homology searches against the RefSeq, TrEMBL, and Clusters of Orthologous Groups (COG) protein databases.

### Genomic data of *Fructobacillus durionis* and *Leuconostoc* spp.

Draft genome sequence of *Fructobacillus durionis* DSM 19113^T^ was obtained from the JGI Genome Portal (http://genome.jgi.doe.gov/) [19] and annotated using MiGAP in the same way as other *Fructobacillus* spp. Annotated genome sequences for nine of the twelve *Leuconostoc* species were obtained from the GenBank or RefSeq databases at NCBI. Of *Leuconostoc* spp., genomic data of *Leuconostoc holzapfelii*, *Leuconostoc miyukkimchii* and *Leuconostoc palmae* were not available at the time of analysis (December 2014) and were not included in the present study. When multiple strains were available for a single species, the most complete one was chosen. GenBank accession numbers of the strains used are listed in Table 1.

**Table 1:** General genome characteristics of the strains analyzed

Strains	Genome status^a^	Source	INSD/SRA accession no.	Size	No. of CDS	%G + C	GC3	Completeness^c^	Contamination^c^
*Fructobacillus fructosus* NRIC 1058^T^	D	Flower	BBXR01000000	1.49	1437	44.6	46.4	93.62	0
*Fructobacillus durionis* DSM 19113^T^	D	Fermented fruit	JGI^b^	1.33	1221	44.7	47.4	94.98	0.57
*Fructobacillus ficulneus* JCM 12225^T^	D	Fig	BBXQ01000000	1.55	1397	43.9	44.6	92.79	0.48
*Fructobacillus pseudoficulneus* DSM 15468^T^	D	Fig	BBXS01000000	1.41	1312	44.5	45.9	95.14	0.48
*Fructobacillus tropaeoli* F214-1^T^	D	Flower	BBXT01000000	1.69	1572	44.2	45.7	94.98	0.24
*Leuconostoc mesenteroides* ATCC 8293^T^	C	Fermenting olives	CP000414-15	2.08	2045	37.7	30.1	100	0
*Leuconostoc carnosum* JB16	C	Kimchi	CP003851-55	1.77	1696	37.1	27.9	99.04	0.6
*Leuconostoc citreum* KM20	C	Kimchi	DQ489736-40	1.90	1849	38.9	31.3	99.52	0
*Leuconostoc fallax* KCTC 3537^T^	D	Sauerkraut	AEIZ01000000	1.64	1882	37.5	29.2	97.30	1.16
*Leuconostoc gelidum* JB7	C	Kimchi	CP003839	1.89	1818	36.7	27.6	99.04	0.24
*Leuconostoc inhae* KCTC 3774^T^	D	Kimchi	AEMJ01000000	2.30	2790	36.4	28.6	95.59	5.38
*Leuconostoc kimchii* IMSNU 11154^T^	C	Kimchi	CP001753-58	2.10	2097	37.9	30.1	99.52	0
*Leuconostoc lactis* KACC 91922	D	Kimchi	JMEA01000000	1.69	2076	43.4	41.1	99.04	0.57
*Leuconostoc pseudomesenteroides* 1159	D	Cheese starter	JAUI01000000	2.04	1634	39.0	32.5	99.04	0.16

^a^Genome status: D, draft genome sequence; C, complete genome sequence

^b^Obtained from Integrated Microbial Genomes (IMG) database at the Department of Energy Joint Genome Institute (http://genome.jgi.doe.gov/)

^c^Determined by CheckM

### Quality assessment of the genomic data

The completeness and contamination of the genomic data were assessed by CheckM (Version 1.0.4) [20], which inspects the existence of gene markers specific to the *Leuconostocaceae* family, a superordinate taxon of *Fructobacillus* and *Leuconostoc*.

### Comparative genome analysis and statistical analysis

To estimate the size of conserved genes, all protein sequences were grouped into orthologous clusters by GET_HOMOLOGUES software (version 1.3) based on the all-against-all bidirectional BLAST alignment and the MCL graph-based algorithm [21]. The conserved genes are defined as gene clusters that are present in all analyzed genomes (please note the difference from the definition of *specific* genes). The rarefaction curves for conserved and total genes were drawn by 100-time iterations of adding genomes one by one in a random order. From this analysis, two genomes (*L. fallax* and *L. inhae*) were excluded to avoid underestimation of the size of conserved genes, since they contained many frameshifted genes, probably due to the high error rate at homopolymer sites of Roche 454 sequencing technology.

For functional comparison of the gene contents between *Fructobacillus* spp. and *Leuconostoc* spp., CDS predicted in each strain were assigned to Cluster of Orthologous Groups (COG) functional classification using the COGNITOR software [22]. Metabolic pathway in each strain was also predicted using KEGG Automatic Annotation Server (KAAS) by assigning KEGG Orthology (KO) numbers to each predicted CDS [23]. The numbers of genes assigned to each COG functional category were summarized as a table (Table 2). In the present study, *Fructobacillus*-specific genes were defined as those conserved in four or more *Fructobacillus* spp. (out of five) and in two or less *Leuconostoc* spp. (out of nine). *Leuconostoc*-specific genes were defined as those conserved in seven or more *Leuconostoc* spp. and one or less *Fructobacillus* spp.

**Table 2:** Gene content profiles obtained for *Fructobacillus* spp. and *Leuconostoc* spp.

	*F. fructosus* NRIC 1058^T^	*F. durionis* DSM 19113^T^	*F. ficulneus* JCM 12225^T^	*F. pseudoficulneus* DSM 15468^T^	*F. tropaeoli* F214-1^T^	*L. mesenteroides* ATCC 8293^T^	*L. carnosum* JB16	*L. citreum* KM20	*L. fallax* KCTC 3537^T^	*L. gelidum* JB7	*L. inhae* KCTC 3774^T^	*L. kimchii* IMSNU 11154^T^	*L. lactis* KACC 91922	*L. pseudomesenteroides* 1159
[C] Energy production and conversion	40	34	41	36	43	69	49	66	39	67	50	68	56	61
[D] Cell cycle control, cell division, chromosome partitioning	35	36	41	37	43	37	33	40	24	33	23	45	30	38
[E] Amino acid transport and metabolism	112	106	159	137	160	192	152	129	110	136	116	179	139	152
[F] Nucleotide transport and metabolism	64	61	77	74	73	91	88	85	71	88	78	97	82	100
[G] Carbohydrate transport and metabolism	61	61	69	63	74	168	123	155	80	172	138	156	120	162
[H] Coenzyme transport and metabolism	51	49	54	49	64	91	73	80	52	72	64	98	78	78
[I] Lipid transport and metabolism	40	43	44	43	51	62	56	71	40	71	59	64	58	57
[J] Translation, ribosomal structure and biogenesis	180	175	188	180	190	193	191	185	162	193	166	198	186	191
[K] Transcription	93	84	89	87	115	133	128	129	93	150	132	153	100	151
[L] Replication, recombination and repair	110	86	97	86	115	110	100	105	57	92	95	119	96	125
[M] Cell wall/membrane/envelope biogenesis	84	77	73	74	84	110	92	105	81	98	75	102	93	94
[N] Cell motility	10	7	6	4	11	11	12	14	7	12	5	17	13	12
[O] Posttranslational modification, protein turnover, chaperones	46	37	47	40	49	63	59	59	39	54	44	67	46	58
[P] Inorganic ion transport and metabolism	49	48	51	54	54	81	70	77	46	61	56	83	63	70
[Q] Secondary metabolites biosynthesis, transport and catabolism	10	7	12	9	12	18	10	13	10	11	12	11	15	15
[R] General function prediction only	67	55	78	67	85	99	83	87	64	89	77	103	79	95
[S] Function unknown	111	100	90	94	114	133	109	122	95	116	108	124	107	118
[T] Signal transduction mechanisms	31	27	36	29	36	60	49	55	46	48	44	60	51	58
[U] Intracellular trafficking, secretion, and vesicular transport	15	12	11	15	24	12	15	11	12	15	10	14	14	12
[V] Defense mechanisms	34	23	37	37	26	35	37	35	24	47	43	52	35	59
[X] Mobilome: prophages, transposons	44	12	26	9	33	27	21	42	18	12	51	43	38	58

The Mann–Whitney *U* test was applied to compare genome features and gene contents of *Fructobacillus* spp. and *Leuconostoc* spp. The *p* value of 0.05 was considered statistically significant. Statistical analysis was performed using IBM SPSS Statistics for Windows (Version 21.0. Armonk, NY: IBM Corp.).

### Phylogenetic analysis

Orthologous clusters that were conserved among all *Fructobacillus* spp., all *Leuconostoc* spp. and *Lactobacillus delbrueckii* subsp. *bulgaricus* ATCC 11842 (as the outgroup) were determined by GET_HOMOLOGUES as described above. For phylogenetic reconstruction, 233 orthologs that appeared exactly once in each genome were selected. The amino acid sequences within each cluster were aligned using MUSCLE (version 3.8.31) [24]. Poorly-aligned or divergent regions were trimmed using Gblocks [25], and conserved regions were then concatenated using FASconCAT-G [26]. A partitioned maximum likelihood analysis was performed to construct the phylogenetic tree with RAxML (version 8.1.22) [27] using the best-fit evolutionary models predicted for each alignment by ProtTest [28]. The number of bootstrapping was 1,000 replicates.

### Polysaccharides production and reaction to oxygen

Polysaccharides production from sucrose were determined by the methods as described previously [29]. Briefly, the strains were inoculated on agar medium containing sucrose as sole carbon source and incubated aerobically at 30 °C for 48 h.

To study reaction to oxygen on growth, the cells were streaked onto GYP agar [8], which contained D-glucose as the sole carbon source, and cultured under anaerobic and aerobic conditions at 30 °C for 48 h as described previously [4]. The anaerobic conditions were provided by means of a gas generating kit (AnaeroPack, Mitsubishi Gas Chemical, Japan). These studies were conducted for the type strains of five *Fructobacillus* species, *Leuconostoc mesenteroides* subsp. *mesenteroides* NRIC 1541^T^, *Leuconostoc citreum* NRIC 1776^T^ and *Leuconostoc fallax* NRIC 0210^T^.

### Data deposition

Annotated draft genome sequences of *F. fructosus* NRIC 1058^T^, *F. ficulneus* JCM 12225^T^, *F. pseudoficulneus* DSM 15468^T^ and *F. tropaeoli* F214-1^T^ were deposited to the DDBJ/EMBL/GenBank International Nucleotide Sequence Database with accession numbers BBXR01000000, BBXQ01000000, BBXS01000000 and BBXT01000000, respectively. Unassembled raw sequence data were also deposited to the database with accession number DRA004155. The phylogenetic tree and associated data matrix for Fig. 6 are available at TreeBASE (Accession URL: http://purl.org/phylo/treebase/phylows/study/TB2:S18090).

## Results and discussion

### General genome features of *Fructobacillus* spp. and *Leuconostoc* spp.

**Fig. 1:**
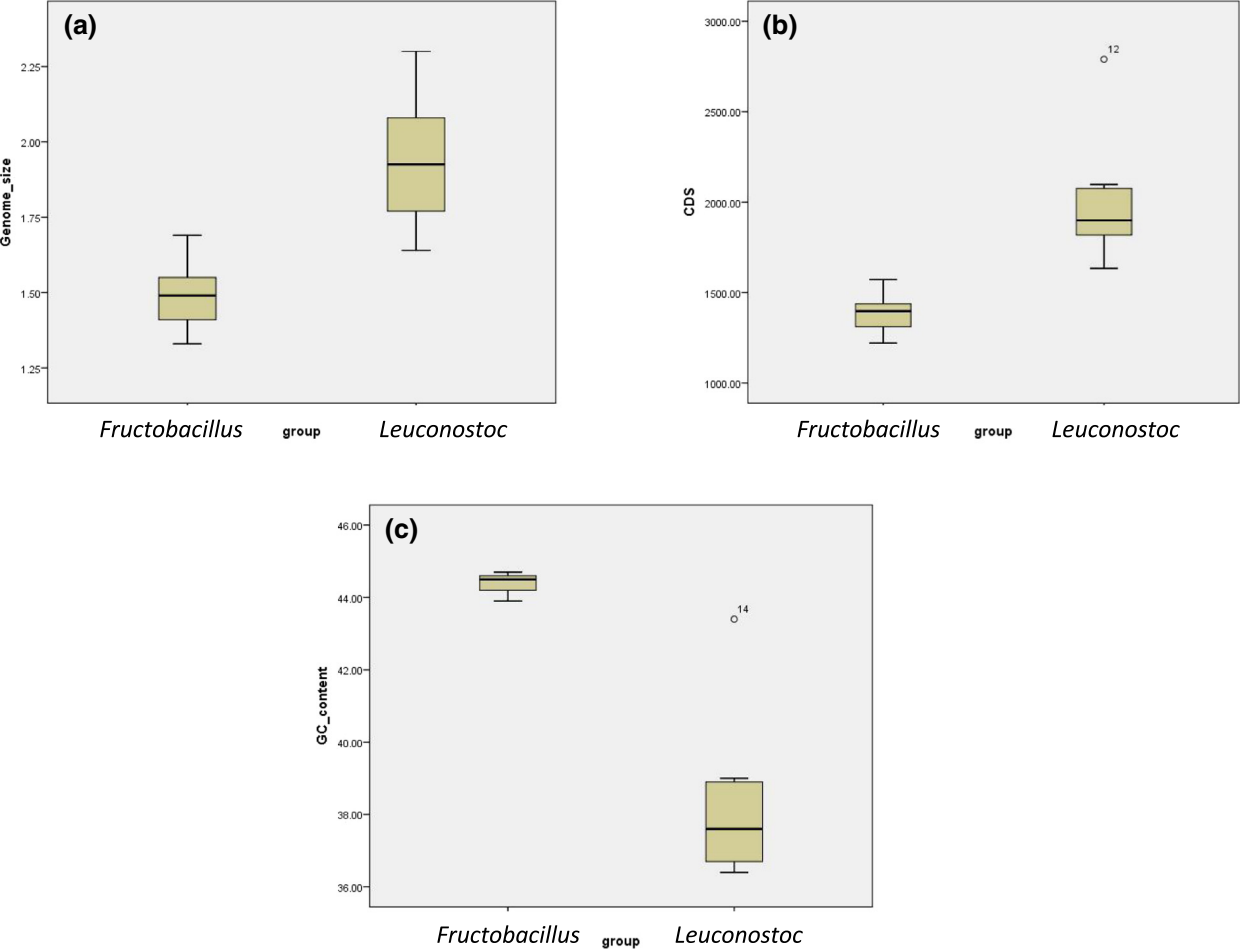
Genome sizes (**a**), number of CDSs (**b**) and GC contents (**c**) in *Fructobacillus* spp. and *Leuconostoc* spp. The line in the box represents the median, with lower line in the 25 % border and the upper line the 75 % border. The end of the upper vertical line represents the maximum data value, outliers not considered. The end of the lower vertical line represents the lowest value, outliers not considered. The separate dots indicate outliers

Draft genome sequences of four *Fructobacillus* spp. were determined by the Illumina Genome Analyzer II system. The sequence coverage of *F. fructosus* NRIC 1058^T^, *F. ficulneus* JCM 12225^T^, *F. pseudoficulneus* DSM 15468^T^ and *F. tropaeoli* F214-1^T^ were 329-, 55-, 90-, and 513-fold, respectively. Genome sequences of nine *Leuconostoc* spp. and *Fructobacillus durionis* were obtained from public databases (see Methods). The genome features of the strains used in the present study are summarized in Table 1. The genome sizes of *Fructobacillus* ranged from 1.33 to 1.69 Mbp (median ± SD, 1.49 ± 0.30 Mbp) and are significantly smaller than those of *Leuconostoc* (*p* < 0.001), 1.69 to 2.30 Mbp (median ± SD, 1.94 ± 0.21) (Fig. 1a). Accordingly, *Fructobacillus* strains contain significantly smaller numbers of CDSs than *Leuconostoc* strains (median ± SD, 1387 ± 132 vs 1980 ± 323, *p* < 0.001) (Fig. 1b). The DNA G + C contents of both species are also significantly different (*p* < 0.001): median ± SD is 44.4 % ± 0.30 % in *Fructobacillus* and 38.1 % ± 2.05 % in *Leuconostoc* (Fig. 1c). The difference in G + C contents is caused by the composition at the third codon (GC3): 46.0 % ± 1.02 % in *Fructobacillus* and 30.9 % ± 4.12 % in *Leuconostoc*. The low GC3 value in *Leuconostoc* spp. shows a good contrast with the high GC3 value in *Lactobacillus delbrueckii* subsp. *bulgaricus* [11]. In *L. delbrueckii* subsp. *bulgaricus*, the changes in GC3 are attributed to ongoing evolution [11], and similar selection pressure might be responsible here. Overall, these distinct genomic features strongly support the reclassification of *Fructobacillus* spp. from the genus *Leuconostoc*.

Since most of the genomes analyzed in this study were in draft status, quality assessment of the genomes was conducted using CheckM. The average completeness values for *Fructobacillus* and *Leuconostoc* genomes were 94.3 and 98.7 %, respectively (Table 1). Except for the genome of *L. inhae*, which exhibited the contamination value of 5.4 %, all genomes satisfied the criteria required to be considered a near-complete genome with low contamination (≥90 % completeness value and ≤ 5 % contamination value) [20]. The lower completeness values for *Fructobacillus* genomes might be attributable to insufficiency of the reference gene markers used by CheckM, for which the genomic data of *Fructobacillus* spp. were not reflected at the time of writing this paper (December 2014), rather than the lower quality of these genomes. In addition, the lower completeness may indicate specific gene losses in the genus *Fructobacillus* since the closer investigation of CheckM results showed that seven gene markers were consistently absent among five *Fructobacillus* genomes while on average, 14.6 markers were absent out of 463 *Leuconostocaceae*-specific gene markers.

### Conserved genes in *Fructobacillus* spp. and *Leuconostoc* spp.

**Fig. 2:**
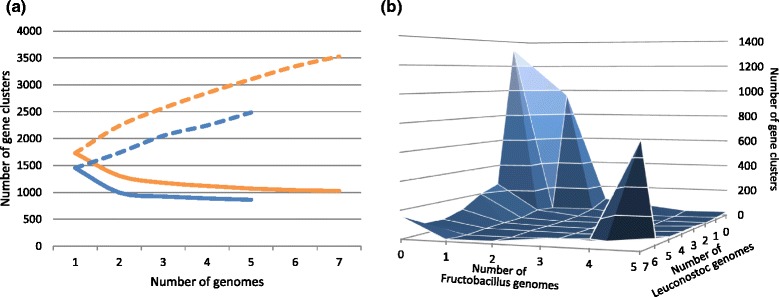
Conserved genes and pan-genome of *Fructobacillus* and *Leuconostoc*. **a** Estimation of the numbers of conserved genes and pan-genome for *Fructobacillus* (blue) and Leuconostoc (orange). Solid lines represent conserved genes and dashed lines represent pan-genomes as a function of the number of genomes added. The medium of 100 random permutations of the genome order is presented. **b** Distribution of gene clusters present in *Fructobacillus* and *Leuconostoc*. Horizontal axes represent the numbers of genomes in each genus. Vertical axes show the numbers of gene clusters present in the given number of genomes

The numbers of conserved genes in the nine genomes of *Leuconostoc* and five genomes of *Fructobacillus* were estimated as 1,026 and 862, respectively. They account for 52 % and 62 % of average CDS numbers of each genus (Fig. 2a). The difference in the average CDS numbers reflects their genomic history including ecological differences between the two genera. A previous study also reported 1162 conserved genes in three genomes of *Leuconostoc* species [30]. The smaller number and the higher ratio of fully conserved genes in *Fructobacillus* spp. is probably due to a less complex and consistent habitat with specific sugars only, such as fructose. It is a major carbohydrate found in habitats of *Fructobacillus* spp., e.g. flowers, fruits and associated insects. On the other hand, *Leuconostoc* spp., that are usually seen in wide variety of habitats, including gut of animals, dairy products, plant surfaces, or fermented foods and soils, possess a larger number of conserved genes. Figure 2b shows the distribution of gene clusters in two genera. The frontmost peak (721 gene clusters) represents conserved genes that are shared by both *Leuconostoc* and *Fructobacillus* spp. Genus-specific conserved genes are indicated as leftmost and right peaks in Fig. 2b. The leftmost peak (159 gene clusters) represents genes that are present in all *Leuconostoc* genomes, but absent in all *Fructobacillus* genomes, and the right peak (24 gene clusters) represents *vice versa*. The much smaller peak of the right compared to that of the left indicates that *Fructobacillus* spp. have lost more genes or have acquired less genes than *Leuconostoc* spp. during diversification after they separated into two groups. In addition, the number of gene clusters located near the center of the figure was small, which indicates that the exchange of genes between the two genera is not frequent and that they share distinct gene pools. This supports the validity of the classification of *Fructobacillus* as a distinct genus [8].

### Comparison of gene contents between *Fructobacillus* spp. and *Leuconostoc* spp.

**Fig. 3:**
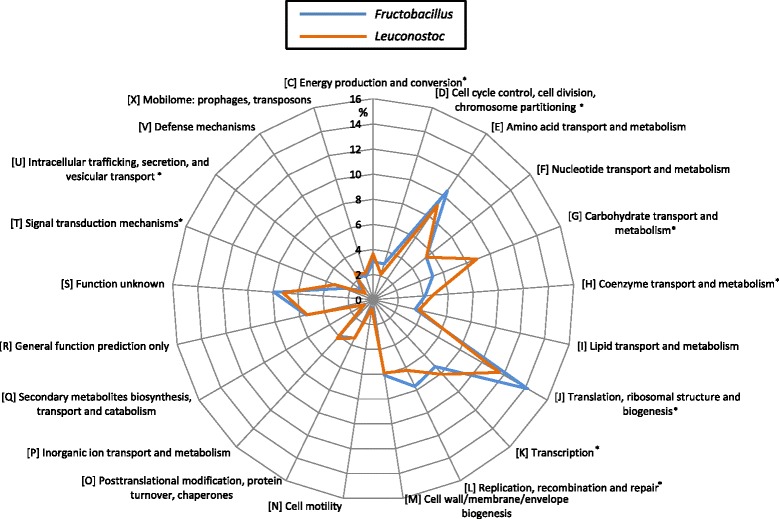
Comparison of ratio (%) of gene content profiles obtained for the genera *Fructobacillus* and *Leuconostoc.* The Mann–Whitney *U* test was done to compare *Fructobacillus* spp. and *Leuconostoc* spp., and significant differences (*P <* 0.05) are denoted with an asterisk (*)

The identified genes were associated with COG functional categories by COGNITOR software at the NCBI. The sizes of COG-class for each strain are summarized in Table 2, and for each genus in Additional file 1: Figure S1. In addition, ratio of genes assigned in each COG category against the total number of genes in all COGs were determined for each genus and shown in Fig. 3. *Fructobacillus* spp. have less genes for carbohydrate transport and metabolism compared to *Leuconostoc* spp. (Class G in Fig. 3 and Additional file 1: Figure S1): Class G ranked 9^th^ largest in *Fructobacillus* whereas it ranked 3^rd^ in *Leuconostoc*. Similarly, the number of genes in Class C (energy production and conversion) was significantly less in *Fructobacillus* spp. than in *Leuconostoc* spp., suggesting that energy systems in *Fructobacillus* spp. are much simpler than those in *Leuconostoc* spp. The smaller number of CDS and conserved genes in *Fructobacillus* spp. could have resulted from metabolic reduction caused by scarce availability of carbohydrates other than fructose.

When compared based on the ratio of genes (Fig. 3), Class D (cell cycle, cell division and chromosome partitioning), Class J (translation, ribosomal structure and biogenesis), Class L (replication, recombination and repair) and Class U (intracellular trafficking, secretion and vesicular transport) were overrepresented in *Fructobacillus* spp. than in *Leuconostoc* spp. However, the numbers of genes classified in the four classes were comparable between the two genera (Additional file 1: Figure S1). The conservation of genes in these classes against the genome reduction may indicate that their functions are essential for re-production, and the class names roughly correspond to housekeeping mechanisms.

To understand gene contents involved in metabolic/biosynthesis pathways in more detail, ortholog assignment and pathway mapping against the KEGG Pathway Database were performed using the KAAS system. The number of mapped genes was significantly less for *Fructobacillus* spp. as compared to *Leuconostoc* spp. (Table 3). Firstly, *Fructobacillus* spp. lack respiration genes. Whereas oxygen is known to enhance their growth [8], the strains have lost genes for the TCA cycle, and keep only one gene for ubiquinone and other terpenoid-quinone biosynthesis (Table 3). Presumably they do not perform respiration and use oxygen only as an electron acceptor. This characteristic is not applicable to certain *Leuconostoc* species: *L. gelidum* subsp. g*asicomitatum* [31]*,* formerly classified as *L. gasicomitatum* [32], has been reported to conduct respiration in the presence of heme and oxygen [33].

**Table 3:** Discriminative pathways between *Fructobacillus* spp. and *Leuconostoc* spp.

	*Fructobacillus* spp.	*Leuconostoc* spp.	
	Mean (SD)^a^	Mean (SD)	*p*
Glycolysis (map00010)	12.2 (0.84)	19.5 (1.72)	0.001
TCA cycle (map00020)	0	4.2 (0.79)	
Pentose and glucuronate interconversions (map00040)	3.2 (1.64)	7.9 (2.80)	0.008
Fructose and mannose metabolism (map00051)	2.8 (0.84)	9.4 (2.12)	0.001
Galactose metabolism (map00052)	5.8 (0.84)	11.6 (2.72)	0.003
Ubiquinone and other terpenoid-quinone biosynthesis (map00130)	1 (0)	7.6 (0.97)	0.001
Oxidative phosphorylation (map00190)	9.2 (0.45)	12.7 (1.57)	0.001
Valine, leucine and isoleucine degradation (map00280)	2 (0)	4.4 (0.84)	0.001
Starch and sucrose metabolism (map00500)	6.4 (1.52)	12.9 (2.28)	0.001
Amino sugar and nucleotide sugar metabolism (map00520)	11.2 (0.45)	19.5 (2.17)	0.001
Pyruvate metabolism (map00620)	12 (1)	19.8 (1.99)	0.001
Carbon metabolism (map01200)	30.6 (3.21)	37.4 (3.20)	0.005
ABC transporters (map02010)	33.8 (3.11)	50.6 (8.34)	0.003
Phosphotransferase system (map02060)	1 (0)	13 (3.13)	0.03

Map numbers shown in parenthesis correspond to the numbers in KEGG

^a^The values indicate means and standard deviations of number of genes used for the pathways

**Fig. 4:**
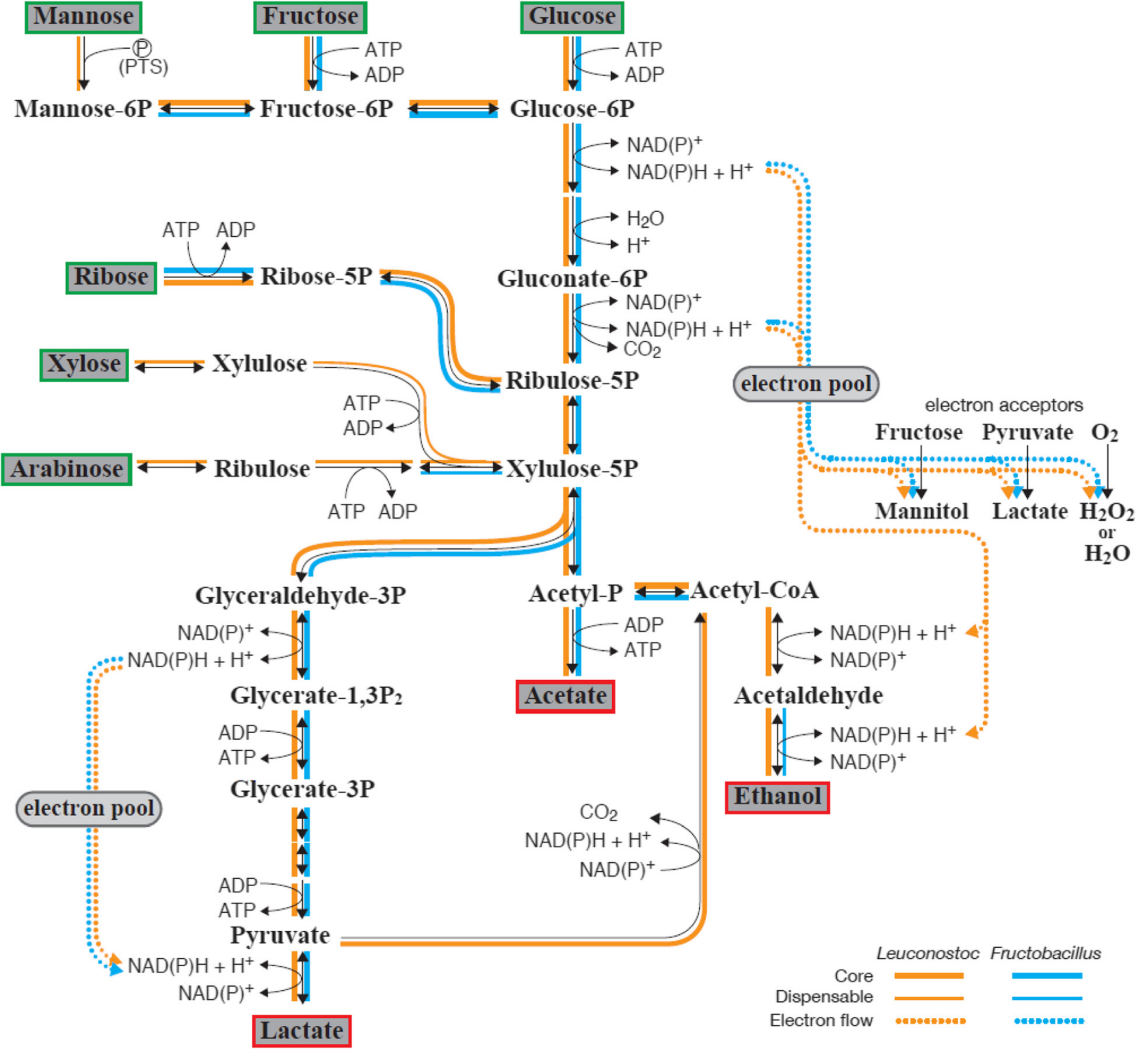
Predicted sugar metabolic pathways in *Fructobacillus* spp. and *Leuconostoc* spp. The orange and blue lines represent the pathways exist in *Leuconostoc* spp. and *Fructobacillus* spp., respectively. The bold lines represent conserved genes among each genus (core) and the narrow lines represent dispensable genes that are exist in some but not all species in each genus. The dotted lines represent electron flow

Secondly, *Fructobacillus* spp. lack pentose and glucuronate interconversions (Table 3). They lost genes for pentose metabolism, unlike other obligately heterofermentative LAB that usually metabolize pentoses [34]. They do not metabolize mannose, galactose, starch, sucrose, amino sugars or nucleotide sugars, either [7, 8]. Moreover, the species possess none or at most one enzyme gene for the phosphotransferase systems (PTS), significantly less than the number of respective genes in *Leuconostoc* spp. (13 ± 3.13, average ± SD). This validates the observation that *Leuconostoc* spp. metabolize various carbohydrates whereas *Fructobacillus* spp. do not [8] (Fig. 4.) However, the genome-based prediction does not always coincide with observed metabolism: *Fructobacillus* species do not metabolize ribose [8], against its metabolic prediction (Fig. 4). The discrepancy is due to an absence of ATP-dependent ribose transporter. On the other hand, some *Leuconostoc* spp. have the transporter and metabolize ribose.

Thirdly, *Fructobacillus* spp. have more genes encoding phenylalanine, tyrosine and tryptophan biosynthesis compared to *Leuconostoc* spp. (Table 3), although this difference is statistically not significant (*p* = 0.165). The difference is mainly due to presence/absence of tryptophan metabolism, and the production of indole and chorismate. This is important to wine lactobacilli [35]. The reason of the sporadic conservation of indole biosynthesis in *Fructobacillus* remains unknown.

### Comparison of genus-specific genes

To further investigate their differences, we defined genes as *Fructobacillus*-specific when they are conserved in four or more *Fructobacillus* species (out of five) and two or less in the nine *Leuconostoc* species. On the other hand, genes are *Leuconostoc*-specific when they are possessed by seven or more *Leuconostoc* species (out of nine) and zero or one in the five *Fructobacillus* species. According to this definition, 16 genes were identified as *Fructobacillus*-specific and 114 as *Leuconostoc-*specific (Additional file 2: Table S1). These numbers are smaller than the numbers of fully conserved genes in each genus (24 for *Fructobacillus* and 159 for *Leuconostoc*)*,* because we defined genus-specific genes after mapping them to the KEGG Orthology (KO) database; genes without any KO entry were excluded from the analysis.

Interestingly the *adh* gene coding alcohol dehydrogenase [EC:1.1.1.1] was characterized as *Fructobacillus*-specific whereas *adhE* gene coding bifunctional acetaldehyde/alcohol dehydrogenase [EC1.2.1.10 1.1.1.1] was characterized as *Leuconostoc*-specific. There was no alternative acetaldehyde dehydrogenase gene in *Fructobacillus*. These results are consistent with our previous study reporting the lack of *adhE* gene and acetaldehyde dehydrogenase activity in *Fructobacillus* spp. [9] and their obligately heterofermentative nature with no ethanol production [6, 8]. No production of ethanol is due to an absence of acetaldehyde dehydrogenase activity, but it conflicts with the NAD/NADH recycling. Therefore, there must be a different electron acceptor in glucose metabolism [4, 6, 9].

**Fig. 5:**
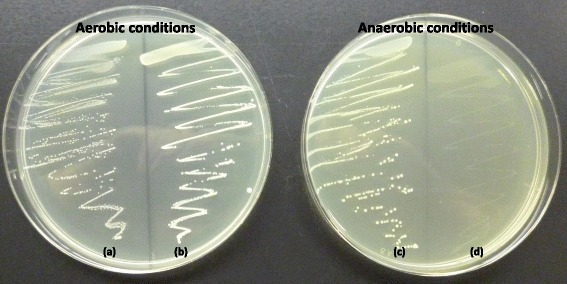
Growth of *L. mesenteroides* NRIC 1541^T^ and *F. fructosus* NRIC 1058^T^ on GYP agar medium under aerobic and anaerobic conditions after incubation for 2 days. *L. mesenteoides* NRIC 1541^T^, a and c; *F. fructosus* NRIC 1058^T^, b and d

NAD(P)H dehydrogenase gene was found as *Fructobacillus-*specific (Additional file 2: Table S1). This is the only gene used for the quinone pool in *Fructobacillus* spp., suggesting that the gene does not contribute to respiration. Rather, it is used for oxidation of NAD(P)H under the presence of oxygen. This helps to keep the NAD(P)/NAD(P)H balance, since their sugar metabolism produces imbalance in NAD(P)/NAD(P)H cycling as described above. Indeed, *Fructobacillus* spp. can be easily differentiated from *Leuconostoc* spp. based on the reaction to oxygen [8]. In our validation study, *Fructobacillus* spp. grew well under aerobic conditions but poorly so under anaerobic conditions on GYP medium (Fig. 5). Presence of oxygen had smaller impacts on growth of *Leuconostoc* spp., but they generated larger colonies under anaerobic conditions than under aerobic conditions.

Genes for subunits of the pyruvate dehydrogenase complex were undetected in the genomes of *Fructobacillus*, but were found in *Leuconostoc. Fructobacillus* also lack TCA cycle genes. This suggests that, in *Fructobacillus*, pyruvate produced from the phosphoketolase pathway is not dispatched to the TCA cycle but metabolized to lactate by lactate dehydrogenase. The lack of pyruvate dehydrogenase complex was also reported in *Lactobacillus kunkeei* [35], which is also a member of FLAB found in fructose-rich environment [4, 36].

The levansucrase gene was also characterized as *Fructobacillus-*specific (Additional file 2: Table S1). The enzyme has been known to work for production of oligosaccharides in LAB [36, 37] and for biofilm production in other bacteria [38]. However, production of polysaccharides was unobserved in *Fructobacillus* spp. when cultured with sucrose. The reason for this discrepancy is yet unknown. Incompetence of sucrose metabolism, including no dextran production, in *Fructobacillus* spp. has been reported [7, 8], and systems to metabolize sucrose, e.g. genes for sucrose-specific PTS, sucrose phosphorylase and dextransucrase, were not detected in their genomes. On the other hand, *L. citreum* NRIC 1776^T^ and *L. mesenteroides* NRIC 1541^T^ produced polysaccharides, possibly dextran. Production of dextran from sucrose in the genus *Leuconostoc* is strain/species dependent [39], and dextransucrase gene was identified in six *Leuconostoc* genomes (out of nine) in this study. A number of genes coding peptidases and amino acids transport/synthesis/metabolism were also found as *Leuconostoc*-specific genes (Additional file 2: Table S1), suggesting that *Leuconostoc* spp. can survive various environments with different amino acid compositions. Several PTS related genes and genes for teichoic acid transport were also characterized as *Leuconostoc*-specific. LAB cells usually contain two distinct types of teichoic acid, which are wall teichoic acid and lipoteichoic acid. The identified genes are involved in biosynthesis of wall teichoic acid in *Bacillus subtilis* [40]. Few studies have been reported for wall teichoic acid in *Leuconostoc* spp. and none in *Fructobacillus* spp.

### Phylogenetic analysis

**Fig. 6:**
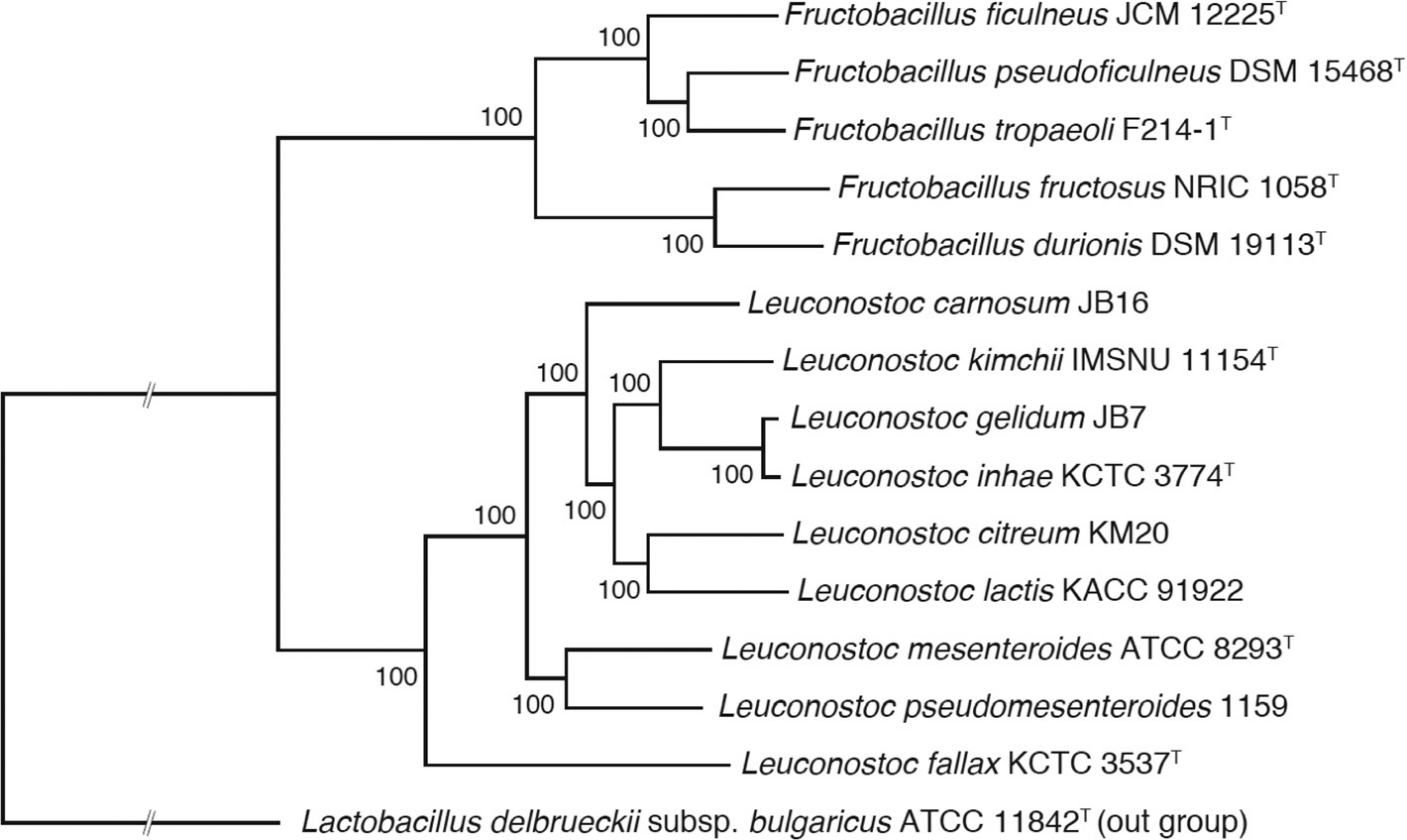
Phlylogenetic tree of *Fructobacillus* spp. and *Leuconostoc* spp. based on the multiple alignments of the 233 conserved genes. The partitioned maximum-likelihood tree constructed using the best-fit evolutionary model clearly separated *Fructobacillus* spp. from *Leuconostoc* spp. The values on the branches are bootstrap support from 1000 rapid bootstrapping replicates. *Lactobacillus delbrueckii* subsp. *bulgaricus* ATCC 11842 was used as an out group

To confirm the phylogenetic relationship between *Fructobacillus* spp. and *Leuconostoc* spp., a phylogenetic tree was produced based on concatenated sequences of 233 orthologous genes which were conserved as a single copy within the tested strains. The tree showed a clear separation of the two genera (Fig. 6), indicating that *Fructobacillus* spp. have distinct phylogenetic position from *Leuconostoc* spp. This agrees well with the previous reports using 16S rRNA gene or house-keeping genes [7, 8].

## Conclusion

Genome-based analysis on conserved genes and metabolic characteristics clearly indicated the distinction between *Fructobacillus* spp. and *Leuconostoc* spp. *Fructobacillus* spp. possess smaller numbers of CDS in smaller genomes compared to *Leuconostoc* spp. This is mainly due to the absence of carbohydrate metabolic systems. Similar genomic characteristics have been reported for *L. kunkeei* [41], a member of FLAB found in fructose-rich environment. Since they are known as poor sugar fermenter in the group of LAB and always inhabit in fructose-rich niches, the characteristics could have resulted from an adaptation to their extreme environments. Niche-specific evolution, usually genome reduction, has been reported for dairy and vaginal LAB [10–12], and the present study reconfirms such niche-specific evolution in FLAB. These findings would be valuable to know a link of diverse physiological and biochemical characteristics in LAB and environmental factors in their habitats.

## Additional files

Additional file 1: Figure S1. Comparison of gene content profiles obtained for the genera *Fructobacillus* and *Leuconostoc*. The Mann–Whitney U test was done to compare *Fructobacillus* spp. and *Leuconostoc* spp., and significant differences (*P* < 0.05) are denoted with an asterisk (*). (PPTX 941 kb) Additional file 2: Table S1. Genus-unique genes for *Fructobacillus* and *Leuconostoc*. (XLSX 15 kb)

## Abbreviations

CDS: protein coding sequences
COG: Clusters of Orthologous Groups
FLAB: fructophilic lactic acid bacteria
KO: KEGG Orthology
LAB: lactic acid bacteria
