# Supplementary material for: Comparative genomics of Fructobacillus spp. and Leuconostoc spp. reveals niche-specific evolution of Fructobacillus spp
Source: BMC Genomics. 2015 Dec 29;16:1117. doi: 10.1186/s12864-015-2339-x (PMC4696137; doi:10.1186/s12864-015-2339-x)
Supplement: Additional file 1: Figure S1. — Comparison of gene content profiles obtained for the genera Fructobacillus and Leuconostoc. The Mann–Whitney U test was done to compare Fructobacillus spp. and Leuconostoc spp., and significant differences (P < 0.05) are denoted with an asterisk (*). (PPTX 941 kb) [file 12864_2015_2339_MOESM1_ESM.pptx]

## Slide 1
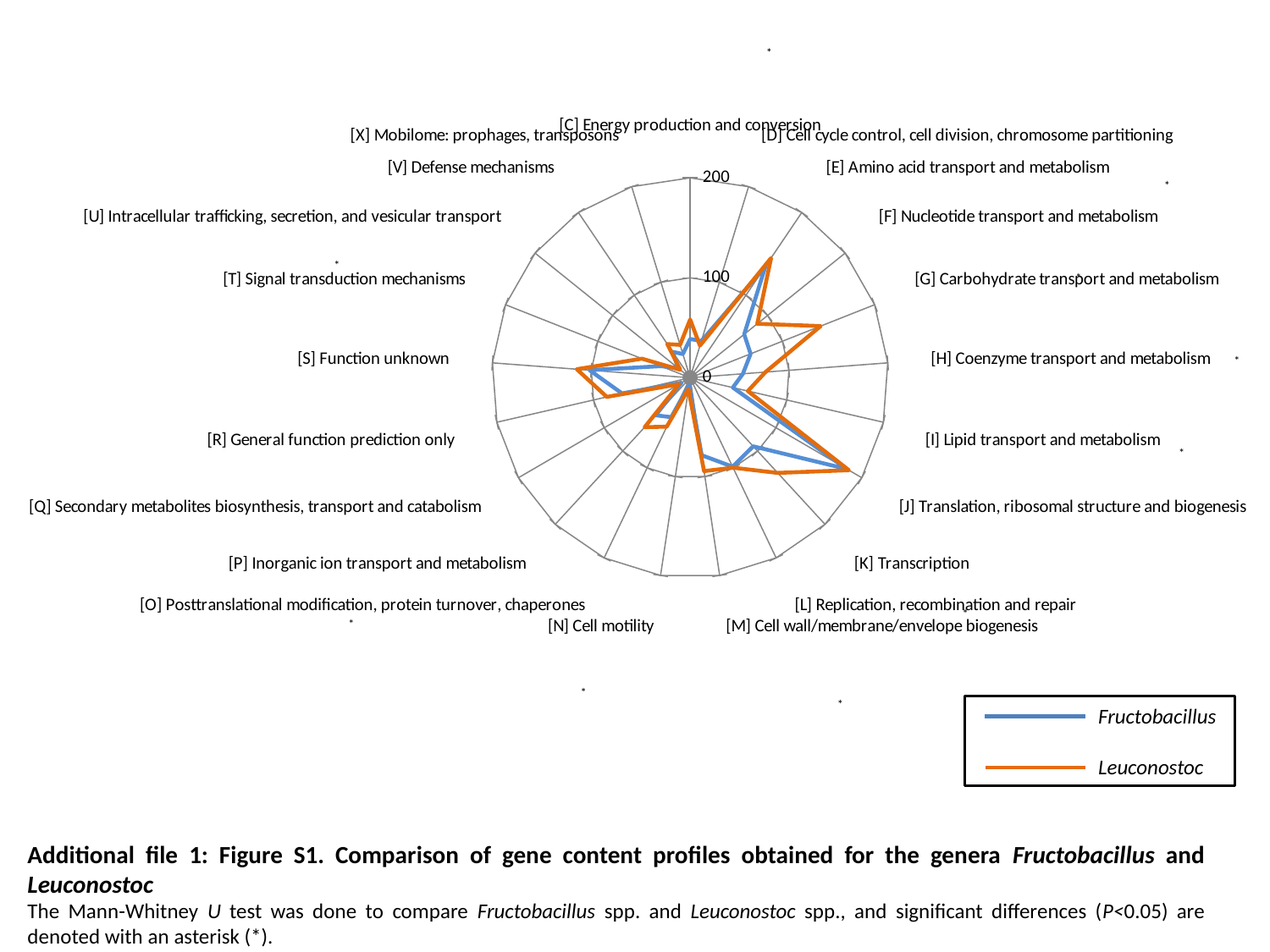

*
### Chart
| Category | Fructobacillus | Leuconostoc |
|---|---|---|
| [C] Energy production and conversion | 38.8 | 58.333333333333336 |
| [D] Cell cycle control, cell division, chromosome partitioning | 38.4 | 33.666666666666664 |
| [E] Amino acid transport and metabolism | 134.8 | 145.0 |
| [F] Nucleotide transport and metabolism | 69.8 | 86.66666666666667 |
| [G] Carbohydrate transport and metabolism | 65.6 | 141.55555555555554 |
| [H] Coenzyme transport and metabolism | 53.4 | 76.22222222222223 |
| [I] Lipid transport and metabolism | 44.2 | 59.77777777777778 |
| [J] Translation, ribosomal structure and biogenesis | 182.6 | 185.0 |
| [K] Transcription | 93.6 | 129.88888888888889 |
| [L] Replication, recombination and repair | 98.8 | 99.88888888888889 |
| [M] Cell wall/membrane/envelope biogenesis | 78.4 | 94.44444444444444 |
| [N] Cell motility | 7.6 | 11.444444444444445 |
| [O] Posttranslational modification, protein turnover, chaperones | 43.8 | 54.333333333333336 |
| [P] Inorganic ion transport and metabolism | 51.2 | 67.44444444444444 |
| [Q] Secondary metabolites biosynthesis, transport and catabolism | 10.0 | 12.777777777777779 |
| [R] General function prediction only | 70.4 | 86.22222222222223 |
| [S] Function unknown | 101.8 | 114.66666666666667 |
| [T] Signal transduction mechanisms | 31.8 | 52.333333333333336 |
| [U] Intracellular trafficking, secretion, and vesicular transport | 15.4 | 12.777777777777779 |
| [V] Defense mechanisms | 31.4 | 40.77777777777778 |
| [X] Mobilome: prophages, transposons | 24.8 | 34.44444444444444 |*
*
*
*
*
*
*
*
*
Fructobacillus
Leuconostoc
Additional file 1: Figure S1. Comparison of gene content profiles obtained for the genera Fructobacillus and Leuconostoc
The Mann-Whitney U test was done to compare Fructobacillus spp. and Leuconostoc spp., and significant differences (P<0.05) are denoted with an asterisk (*).
